# Nanomaterials as Assisted Matrix of Laser Desorption/Ionization Time-of-Flight Mass Spectrometry for the Analysis of Small Molecules

**DOI:** 10.3390/nano7040087

**Published:** 2017-04-21

**Authors:** Minghua Lu, Xueqing Yang, Yixin Yang, Peige Qin, Xiuru Wu, Zongwei Cai

**Affiliations:** 1 Institute of Environmental and Analysis Science, College of Chemistry and Chemical Engineering, Henan University, Kaifeng 475004, Henan, China; yangyixin7375@163.com (Y.Y.); qinpeige@outlook.com (P.Q.); 18337132492@sina.com (X.W.); 2 State Key Laboratory of Environmental and Biological Analysis, Department of Chemistry, Hong Kong Baptist University, Hong Kong, China; yangxueqing1988@hotmail.com

**Keywords:** nanomaterial, laser desorption/ionization, time-of-flight mass spectrometry, small biological molecules, environmental pollutants

## Abstract

Matrix-assisted laser desorption/ionization (MALDI), a soft ionization method, coupling with time-of-flight mass spectrometry (TOF MS) has become an indispensible tool for analyzing macromolecules, such as peptides, proteins, nucleic acids and polymers. However, the application of MALDI for the analysis of small molecules (<700 Da) has become the great challenge because of the interference from the conventional matrix in low mass region. To overcome this drawback, more attention has been paid to explore interference-free methods in the past decade. The technique of applying nanomaterials as matrix of laser desorption/ionization (LDI), also called nanomaterial-assisted laser desorption/ionization (nanomaterial-assisted LDI), has attracted considerable attention in the analysis of low-molecular weight compounds in TOF MS. This review mainly summarized the applications of different types of nanomaterials including carbon-based, metal-based and metal-organic frameworks as assisted matrices for LDI in the analysis of small biological molecules, environmental pollutants and other low-molecular weight compounds.

## 1. Introduction

Matrix-assisted laser desorption/ionization (MALDI), a powerful soft ionization method developed by Tanaka [1] and Karas [2], coupled with time-of-flight mass spectrometry (TOF MS) has become an indispensable analytical tool in complex sample analysis [3,4,5,6,7,8]. Due to its inherent properties including high-throughput, high sensitivity, high tolerance towards salts, fast analysis ability, small sample consumption, simple sample preparation, and no or little fragmentations, over 38,000 related papers have been published in the past three decades. Most of these research papers were focused on large biomolecules (e.g., peptides, proteins and nucleic acids) [9] and synthetic polymers [10]. However, it is difficult to measure small molecules with molecular weight lower than 700 Da because of the interference coming from the conventional organic compound matrices (e.g., α-cyano-4-hydroxycinnamic acid (CHCA), 2,5-dihydroxybenzoic acid (DHB), and sinapic acid (SA)) in low mass region. In the past decades, considerable efforts have been made for developing various techniques and methods to solve the problems of MALDI-TOF MS in the analysis of low molecular weight compounds [11,12,13,14].

Nanomaterials have been widely developed as efficient assisted matrices for LDI profiling small molecules [15,16,17,18]. The history of nanomaterials as matrix for MALDI can be traced to the 1980s, in which cobalt nanopowder mixed with glycerol in organic solvent (e.g., ethanol and acetone) were used to ionization of synthetic polymers and proteins. In 1995, Sunner and co-workers [19] used 2–150 µm graphite particles as matrix to ionization of peptides, proteins, and small molecules. Protonated analytes and abundant alkali cation adducts, as well as dominated interferences from carbon cluster ions (C_n_^+^) were observed at elevated laser powers. In 1999, G. Siuzdak et al. [20] introduced a truly matrix-free strategy for the analysis of biomolecular based on LDI on a porous silicon surface (DIOS), which started a new era for analysis of small molecules with LDI-TOF MS. The method using porous silicon to trap analytes that deposited on the surface, followed by laser irradiation and ionization, reached to femtomole and attomole levels with little or no fragmentation. Moreover, the research group also further explored the surface chemical modification for DIOS as the matrix for MALDI, which enhanced the sensitivity and specificity [21,22].

Compared with traditional MALDI technique that using organic compounds (e.g., CHCA, DHB and SA) as matrix, nanomaterials as matrix for LDI (also called nanomaterial-assisted LDI) has some unique advantages and characteristics in the analysis of small molecules. Firstly, different from the conventional matrices, nanomaterial-assisted LDI usually provides a background-free mass spectrum in the low molecule weight regions (<700 Da) [23]. Secondly, nanomaterial-assisted LDI usually possesses excellent shot-to-shot reproducibility because the co-crystallization process may be avoided that usually produces an inhomogeneous mixture and results in “hot spots” when using traditional matrices [24]. Thirdly, nanomaterials can not only serve as matrix that transfers energy from laser to the analytes, but also act as an adsorbent for clean-up and enrichment of analytes in complex samples, which can low its detection limits in the LDI-MS analysis [25]. Fourthly, nanomaterial-assisted LDI usually exhibits higher signal intensity than that of conventional MALDI after enrichment because the competition of analytes and matrix was eliminated in the process of ionization [26].

Consequently, development of high-throughput, sensitive and reproducible methods for the analysis of small biological molecules and environmental pollutants have attracted more and more attention in recent years. Compared with electrospray ionization (ESI), MALDI has the ability to eliminate contamination during the process of analysis of biological and environmental samples. However, due to the interference in low-molecular weight region of the traditional organic matrices, MALDI was seldom applied in analysis of small biological molecules and environmental pollutants in the past. With the development of various nanomaterials that can be used as assisted matrix, the applications of MALDI in small biological molecules and environmental pollutants have gained more and more attention. In this review, nanomaterials including carbon-based, metal-based and metal organic frameworks (MOF) based materials that were applied as the assisted matrices for LDI for the analysis of small biological molecules, environmental pollutants, and other low-molecular weight compounds, were reviewed, with a focus on publications from 2010 to present.

## 2. Nanomaterial-Assisted LDI Method Development

**Figure 1:**
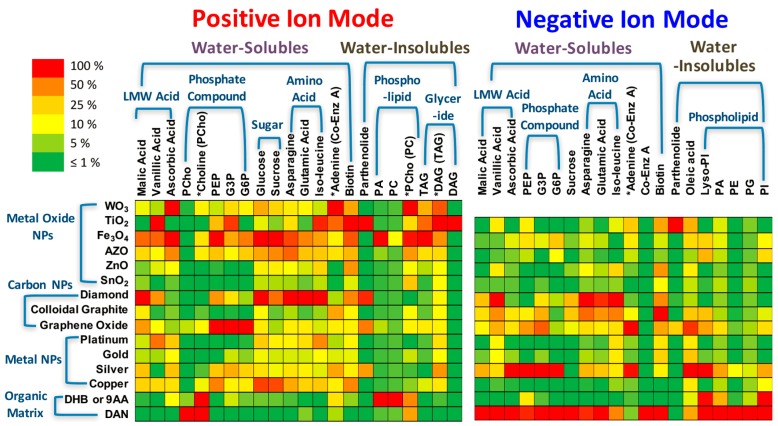
Summary of nanoparticle screening for small molecule metabolite analysis. Ion signals are normalized to the highest ion signal for each analyte and shown as a heat map. WO_3_ NPs have significant matrix background in negative mode and were not used for the final screening. An asterisk indicates a fragment ion with the precursor shown in parentheses. 2,5-dihydroxybenzoic acid (DHB); 9-aminoacridine (9AA); 1, 5-diaminonaphthalene (DAN). DHB and DAN were used for positive ion mode and 9AA and DAN were used for negative ion mode. Reproduced with permission of [27].

The nanomaterials selected as matrix for LDI in the analysis of small molecules is often a key matter of trial and error. Recently, a number of nanomaterials have been utilized in nanomaterial-assisted LDI. As a typical example, a large scale screening of 13 nanoparticles (NPs), including metal oxide NPs (WO_3_, TiO_2_, Fe_3_O_4_, AZO (aluminum-doped zinc oxide), ZnO, SnO_2_), carbon-based NPs (boron doped nanodiamond, colloidal graphite, graphene oxide), and metal NPs (Pt, Au, Ag, Cu), the analysis of two dozen small metabolite molecules was reported by Yagnik et al. [27]. The heat map demonstrated that a large number of NPs showed much higher LDI efficiency than that of traditional organic compounds in positive mode and some NPs showed comparable efficiencies in negative ion mode (e.g., Ag for olefins, Ag and Au for sulfur compounds) (Figure 1). The obtained results indicated that metal oxide and diamond NPs exhibited high LDI efficiency in the positive ion mode, resulting from the good laser absorption efficiency at 355 nm and low thermal conductivity. This whole process is thermally driven, largely independent on the analytes because cationization process begins to occur after gasification of analytes, showing high specificity. Moreover, other NPs showed good specificity both in the positive and negative ion modes, due to the specific affinity of the analytes on the NPs. Therefore, besides chemical interaction, thermal driven desorption plays a significant role in nanomaterial-assisted LDI using the metal oxides NPs.

Due to the inherent advantages, such as unique structure, large surface area, good performance in optical properties, carbon-based nanomaterials including fullerenes [28], nanodiamond [29,30], nanofibers [31], nanohorns, nanotubes, nanodots, and graphene have been considered as a kind of good candidate matrix for LDI to analysis of small molecules since Sunner and co-workers [19] demonstrated that graphite particles with size of 2–150 µm can be served as matrix for LDI-TOF MS.

Carbon nanotubes (CNTs) were firstly utilized as matrix for LDI-TOF MS to analysis of small molecules in 2003 [32]. The hydrophobicity of CNTs makes it difficult to be the homogeneous matrix, resulting to the poor reproducibility, and thus limits the applications of CNTs in the LDI-MS. To overcome it, a series of CNTs-based matrix were developed. CNTs [33] or oxidized CNTs [34,35] were used as matrix to ionization of various small molecules in traditional Chinese medicines (TCMs) and environmental samples, as well as carbohydrates and amino acids. Hsu et al. [36] developed a multiwalled CNT-based LDI-TOF MS approach for quantification of fatty acids in human plasma. Recently, functionalized CNTs were introduced to improve the ionization efficiency of LDI-TOF MS. Carboxylated CNTs were functionalized with SA either covalently or by creating an ionic macro-complex, and were applied to analysis of two low molecular weight polymers [37]. The results demonstrated that CNTs covalently functionalized with SA as a matrix provided higher peak intensities than that of CNTs functionalized by ionic macro-complex formation. The authors [38] also prepared gallic acid or SA functionalized CNTs by covalently attached to the CNTs surfaces with forming an ester bond for analyzing vancomycin, folic acid and Triton^®^ X-100. The obtained mass spectra revealed the assisting surfaces with the ability of transferring energy to the analytes and the presence of carboxyl groups in the structures of CNTs could highly enhance their ionization efficiency.

Carbon dots (CDs) as a newly emerged member of the carbon nanomaterial family has attracted much attention because of their remarkable characteristics, such as chemical inertness, low toxicity and good biocompatibility [39]. Due to the strong adsorption efficiency in ultraviolet light (220–350 nm) range, ultra-small size (2–4 nm) and excellent water solubility, CDs was considered as an ideal matrix of LDI-TOF MS for the analysis of small molecules. Chen et al. [40] demonstrated CDs as matrix for LDI might work in both positive-and negative-ion modes. The method was applied to analyze various low molecular weight compounds with high sensitivity. For instance, the limit of detection (LOD) of octadecanoic acid was 0.2 fmol. Mass spectra with sodium and potassium adducts were detected in positive mode, and deprotonated signals were obtained in negative mode. The established method was applied to quantitatively determination of glucose in serum and uric acid in urine with the large linear range of 0.5–9 nm and 0.1–1.8 nm (*R*^2^ > 0.999), respectively.

Graphene, as a new member of carbon materials, has attracted considerable attention in various fields from the beginning of 2004 [41]. Their unique properties, such as the high surface area (2630 m^2^/g), excellent optical and electrical properties, make it to be one of most excellent matrices for LDI-MS. Graphene was first reported by Dong and coworkers [42] as matrix for LDI-TOF MS. When compared with traditional organic matrices, polar compounds and nonpolar compounds could be ionized in positive mode using graphene as the matrix with high desorption/ionization efficiency. Therefore, graphene as matrix not only avoided the fragmentation of analytes, but also provided good reproducibility, high salt tolerance and low limits of detection.

Lu et al. [43] demonstrated graphene as matrix in both positive and negative modes. In positive ion mode, multiple adduct ions, such as sodium adduct [M + Na]^+^, potassium adduct [M + K]^+^, double sodium adduct [M + 2Na − H]^+^, double potassium adduct [M + 2K − H]^+^, as well as sodium and potassium mixed adduct [M + Na + K − H]^+^ were produced. LDI with graphene in negative ion mode, however, only generated [M − H]^−^ ions. Therefore, in the negative ion mode, graphene can be utilized as the interference-free matrix that is suitable to analyze a series of low molecular weight compounds. Based on that, six kinds of amino acids were analyzed using graphene and compared with CHCA. The obtained data showed CHCA and graphene as the matrix with strong background interference in the positive mode, while the spectrum contained little interference when graphene was used as the matrix in the negative mode with better sensitivity and reproducibility

The mechanization of LDI on graphene oxide (GO) multilayer films for analysis of small molecules was studied by Kim and Min [44]. The GO films with one to ten layers were prepared through layer-by-layer (LBL) assembly cycles with precisely controlled thickness and surface roughness. Results demonstrated optimal film layers and the threshold energy for LDI-TOF MS associated with the chemical structures of the analytes. The influence of size-fractionalized GO on the performance of LDI for analysis of small molecule was investigated by the same group [45]. The authors concluded that large GO sheets (>0.5 µm) were more prone to fragmentation under LDI than that of small GO sheets (<0.5 µm). Therefore, the analysis efficiency of LDI-TOF MS for small molecules was significantly improved by using nanosized GO as a matrix. They [46,47] also prepared GO/MWCNT double layer films and investigated the mechanization of LDI of small molecules. The LDI efficiency was related to thickness, assembly sequence and surface roughness of the hybrid films.

In order to enhance ion efficiency and gain sensitivity, the composites of graphene associated with other materials were explored, which might combine the advantages of both materials. For instance, graphene coated with mesoporous silica (G@SiO_2_) was prepared and utilized as matrix for sensitive and selective determination of environmental pollutants and biomolecules [48]. Compared with graphene or conventional matrices, G@SiO_2_ nanocomposites possessed higher ionization efficiency and sample localization because of silica mesoporosity. Further, a sol-gel derived, porous GO-embedded film as a substrate of L24DI-TOF MS for metabolite fingerprinting of crude samples was reported by Lee and co-workers [49]. A free-interference background spectrum in the low mass region was achieved with GO embedded sol-gel film, which was more practical in metabolite fingerprinting applications, particularly in the negative ion mode.

Bulk graphitic carbon nitride (g-C_3_N_4_), one of the most stable carbon nitride allotrope, with a graphite-like lamellar structure and high surface area, has been investigated in various areas. Recently, Lin et al. [50] used g-C_3_N_4_ nanosheets as an assisted matrix of LDI for analyzing various different low molecular weight compounds. Mass spectra with free background interference and improved signal intensity were obtained for analyzing a series of small molecules including amino acids, nucleobases, peptides, bisphenols, and nitro-polycyclic aromatic hydrocarbons (nitro-PAHs) in the negative mode. The g-C_3_N_4_ nanosheets based negative ion LDI-TOF MS method was successfully applied to detection of 1-nitropyrene (1-NP) in sewage with the corresponding detection limit was lowered to 1 pmol.

Besides carbon-based nanomaterials, metal-based nanomaterials are agood candidate as highly efficient matrices for LDI-MS for the analysis of small molecules. Among various metal-or metal oxide-based nanomaterials (e.g., Au [51,52,53,54,55], Ag [56], Pt [57,58,59], TiO_2_ [60,61,62], ZnO [63], CdS [64], and HgTe [65] NPs) that explored as matrices for LDI-TOF MS, TiO_2_ and gold NPs (Au NPs) attracted much more attention because their intrinsic advantages, such as large surface area, strong absorption efficiency in UV-visible region, high chemical stability, easy modification and preparation.

Nowadays, TiO_2_ nanomaterials have attracted tremendous interest in photocatalysis, sensor technology, optical coating and pigments owing to their attractive physical and chemical properties [66]. Generally, as we all known, TiO_2_ nanomaterials own three different crystal structures including rutile, anatase, and brookite. Due to strong absorption in the UV range and the ability to transfer energy to analytes rapidly, TiO_2_ nanomaterials have been considered as one of the most promising matrices for LDI-TOF MS to analysis of small molecules. Castro et al. [67] investigated the performance of TiO_2_ with different types (anatase, rutile and their mixture) as matrix for LDI-TOF MS. Caffeine, PEG200, chloroaniline and quercetin could be ionized with TiO_2_ anatase. TiO_2_ pure forms, particularly anatase was proved to be a more useful matrix than their mixture because the materials with pure phase are more stabile under laser irradiation.

Sonderegger et al. [68] reported a method for analysis of small molecules by using a conventional MALDI steel target coated with a 50-nm TiO_2_ layer. The layer without pores or islands was prepared by electron beam evaporation. Compared with TiO_2_ nanopowder matrix, the signal intensity was improved more than 30-fold for the analysis of PEG200 with the prepared TiO_2_-coated target. Mass spectrum showed strong cationization signals with sodium and potassium for analysis of five different sugars. The extract from *Cynara scolymus* leaves was analyzed by the developed method and results demonstrated that all three of the flavonoids were detected with their protonated ions, whereas all other compounds were ionized as [M + Na]^+^ and [M + K]^+^.

TiO_2_ nanocrystals with various shapes and sizes, such as the colloidal TiO_2_ NPs, TiO_2_ prolate nanospheroids (TiO_2_ PNSs), and TiO_2_ nanotubes (TiO_2_ NTs), were investigated as matrix of LDI-TOF MS for quantitative analysis of small molecules [69,70]. Results showed that the applicability of individual TiO_2_ nanocrystals depended on the analyte, such as all TiO_2_ nanocrystals, regardless of their shape, have great potential for the detection and determination of steroid hormones, amino acids and saccharides with good detection limits. In generally, the best reproducibility was obtained with the larger nanocrystals, TiO_2_ PNSs and TiO_2_ NTs, making them good candidates for the quantitative determination of small molecules. However, for analysis of citric acid, dexasone, vitamins E and A, PNSs provided the highest sensitivity and reproducibility [71].

Due to simple sample preparation, strong ionization efficiency, excellent shot-to-shot reproducibility, and high salt tolerance, Au NPs has achieved widely application in analysis of small molecules by LDI-MS since it was introduced as matrix by Mclean et al. in 2005 [72]. Recently, Abdelhamid and Wu [73] reviewed the applications of Au NPs assisted LDI-MS in various analytes including simple molecules and intact cells. However, Au NPs as a LDI matrix often produces many Au cluster peaks, which not only suppress the ionization of the analytes, but also result in complex mass spectrum. Moreover, Au NPs usually provide poor reproducibility because Au colloids may become inhomogeneously distribution on the target plate. To overcome above drawbacks, various functionalized Au NPs were prepared and investigated as matrix for LDI-MS for analysis of small molecules in recent years. An on-chip patterned Au NPs microarray functionalized with a nanoscale silicate coating was prepared by LBL and was used a platform for analysis of small molecules with LDI-TOF MS [54]. The chip-based calcinated AuNP microarray could be regenerated under mild conditions with no deterioration of performance over many cycles, which not only minimized sample preparation, but also large reduced material and reagent cost.

**Figure 2:**
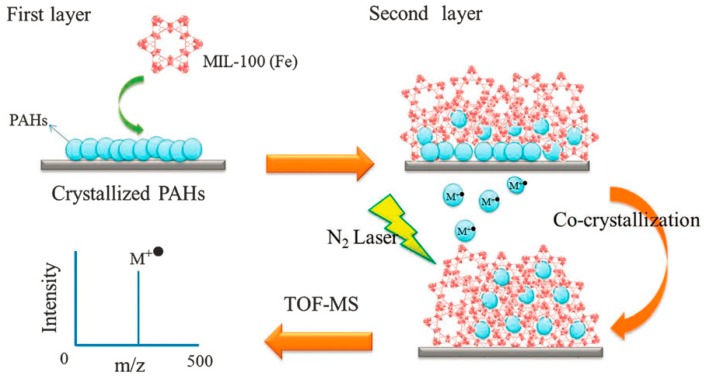
Laser desorption/ionization-mass spectrometry (LDI-MS) process using porous metal organic frameworks (MOFs) as matrices. Reproduced with permission of [75].

Metal organic frameworks (MOFs) are a new type of hybrid crystalline nanomaterials that are constructed from clusters of metal ions and organic ligands [74]. Because of their unique properties including large surface area, high porosity, and tunable pore sizes, MOFs have been utilized in gas storage, separation, catalysis, magnetism, and adsorption. Moreover, MOFs could provide high absorption efficiency in the UV-visible range, which meets the requirement of matrix for LDI. As a result, MOFs as a matrix for LDI-TOF MS for the analysis of small molecules was first introduced by Huang’s group in 2013 [75]. In this work, several porous MOFs (MIL-100 (Fe), MIL-100 (Cr), MIL-100 (Al), MIL-101 (Cr), DUT-4 (Al), DUT-5 (Al) and CYCU-3 (Al)) were synthesized and used as matrices for LDI-MS (Figure 2). The properties of above MOFs were evaluated as matrices by using five PAHs (anthracene, pyrene, benzo[α]anthracene (BaA), chrysene, benzo[α]pyrene (BaP) as test analytes. Results showed that free background interference obtained from cage-type MOFs MIL-100 and MIL-101 (Cr) and very low background interference from tunnel-type MOFs (DUT-4 (Al), DUT-5 (Al) and CYCU-3 (Al)). The influence of different metal ion connectors on the performance including MIL-100 (Fe), MIL-100 (Cr), MIL-100 (Al), MIL-101 (Cr) as matrix were investigated and the results revealed each MIL-100 could provide the absence of background signals. However, the different metal types influenced the PAHs intensity and signal reproducibility. Cage-type 3D MIL-100 (Fe) provided the best shot-to-shot reproducibility. This nanomaterial was reported to serve as matrix for LDI-MS for the detection of biomolecules by the same group [76].

ZIF-7, ZIF-8 and ZIF-90 that are MOFs serving as both the sorbent and matrix of LDI-TOF MS for the enrichment and analysis of bisphenols and environmental pollutants were studied by Yang et al. [24]. ZIF-7 and ZIF-8 were more suitable as a matrix than ZIF-90 because of the strong background interference and relatively larger size of ZIF-90. Although ZIF-7 and ZIF-8 had similar size and structure, ZIF-8 showed better performance including high signal intensity and low background interference because of the large surface area of ZIF-8 (1268 m^2^/g) compared with that of ZIF-7 (247 m^2^/g), which might probably lead to higher laser energy absorption and transfer efficiency.

Gu et al. [77] first reported an inhomogeneous bulk MOFs of 2-D Zn_2_ (bim)_4_ nanosheets derived from ZIF-7 as the matrix in the nanomaterial-assisted LDI-MS for the analysis of amino acids, nucleobases, neurotransmitters, hormones and pollutant molecules. This matrix not only tested the analytes with clean background and good reproducibility, but also suffered from the high salt concentration up to 1000 mM of NaCl and NH_4_HCO_3_ and that opens up an avenue for the bulk MOFs as the matrix.

Chen et al. [78] introduced a method to design and prepare MOFs as adsorbents and matrices according to the structure of ligands and common matrices. 2,5-Pyridinedicarboxylic acid and 2,5-dihydroxyterephthalic acid which have similar chemical structure with traditional organic matrices of 2-picolinic acid and DHB were selected as ligands for the synthesis of MOFs. Two Zr(IV)-based MOFs materials (UiO-66-PDC and UiO-66-(OH)_2_) were prepared and utilized as matrices for LDI of low molecular weight compounds. Mass spectra with low background interference, strong signal intensity and excellent shot-to-shot reproducibility were achieved. UiO-66-(OH)_2_ demonstrated great potential application in quantitative analysis of glucose and pyridoxal 5′-phosphate. Particularly, the material exhibited high affinity toward phosphoric groups by forming Zr-O-P bonds, which made the method have high selectivity and sensitivity to phosphorpeptides.

The composites of MOFs were also investigated in nanomaterial-assisted LDI-MS analysis. For instance, Lin et al. [79] recently reported ZIF-8 coated magnetic nanocomposites (Fe_3_O_4_@ZIF-8 MNCs) was served as absorbent and matrix for sensitive analysis of small molecules, which combined the merits of MOFs with Fe_3_O_4_. The as-prepared Fe_3_O_4_@ZIF-8 MNCs possessed large specific surfaces, excellent thermal stability, superparamagnetic behavior and strong UV-visible adsorption near the laser wavelength. The results demonstrated that Fe_3_O_4_@ZIF-8 MNCs could provide free background interference, good salt tolerance, and enhanced signal intensity in the negative ion mode.

Carbonized MOFs (cMIL-53 and cCYCU-3), a kind of carbon materials derived from MOFs with properties of high surface area, large pore volume, and lower heat capacity compared with conventional carbon-based materials, as well as carry hydrophilic nature without surface modification, was proposed as the matrix for LDI-TOF MS [80]. Compared with the pristine MOFs and other nanoporous carbons, carbonized MOFs exhibit much higher efficiency desorption/ionization of polar and nonpolar small molecules without any matrix interferences in the low molecular weight region. The carbonized MOFs worked under reflectron positive ion mode for analysis of carbohydrates, peptides, phthalate esters and PAHs except phenolic acids under reflectron negative ion mode.

## 3. Nanomaterial-Assisted LDI for the Analysis of Small Biological Molecules

**Figure 3:**
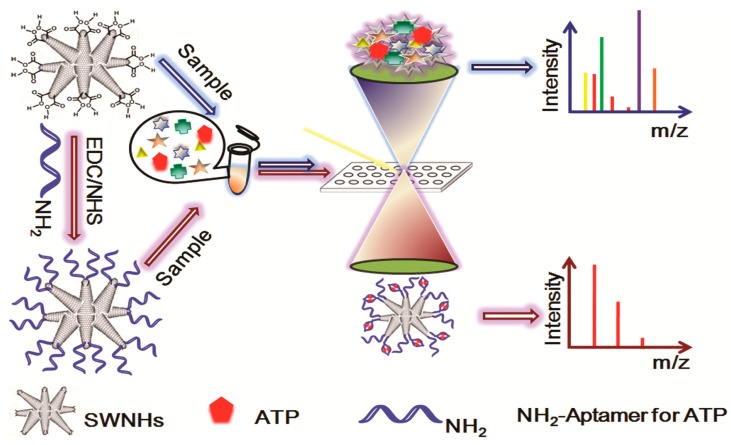
Schematic diagram of aptamer modification of single-walled carbon nanohorns (SWNHs) and negative ion laser desorption/ionization-time-of-flight mass spectrometry (LDI-TOF MS) analysis using functional SWNHs as matrix. Reproduced with permission of [81].

Utilizing the above nanomaterials as the matrix, a variety of small molecules (<700 Da) can be analyzed in the nanomaterial-assisted LDI-MS with free background interference, including small biological molecules, environmental pollutants and other small molecules. Small biological molecules are a type of low-molecular weight (<700 Da) organic compounds that may assist to regulate a biological process. Various nanomaterials have been synthesized and applied in nanomaterial-assisted LDI for analysis of the biological molecules. For instance, Ma et al. [81] prepared single-walled carbon nanohorns (SWNHs) and used as the matrix for LDI-TOF MS for the analysis of various biomolecules including amino acids, peptides and fatty acids. To further improve the selectivity and sensitivity, aptamers-modified SMNHs (Apt-SWNHs) was synthesized by carboxylic groups conjugated with adenosine triphosphate (ATP) aptamers via forming the amide between the –COOH group on the SWNHs and the –NH_2_ moiety of the aptamer. The as-prepared functional materials could act as both an affinity extraction and detection platform for simultaneous capture, enrichment and ionization of ATP from complex biological samples (Figure 3). The LOD of the developed Apt-SWNHs based method was greatly improved (1.0 µM) for the analysis of ATP in complex biological media. Calandra et al. [82] utilized carbon nanohorns as a matrix for LDI-TOF MS detection of irinotecan, sunitinib and 6-α-hydroxy-paclitaxel.

CDs were demonstrated as a useful matrix for LDI for analyzing small biological molecules. Khan et al. [83] utilized CDs as matrix of LDI-TOF MS for the analysis of serotonin, glutamic acid and dopamine hydrochloride with LODs of 3, 5 and 8 nM, respectively. Wang et al. [84] prepared N,S-doped CDs (N,S-CDs) and used as the matrix for the analysis of small molecules with LDI-TOF MS. Compared with conventional CHCA, 9-Aminoacridine (9-AA) and N-CDs matrices, N,S-CDs provided high signal intensity without matrix interference, especially in negative ion mode, as well as good salt tolerance and reproducibility. The as-prepared N,S-CDs was further applied to quantitative determination of endogenous glucose. Compared with N-CDs, the doped S in N,S-CDs played an important role in the highly efficient desorption/ionization process. The oxygen content of N,S-CDs was greatly decreased when the S was doped. On the other hand, the pyridinic nitrogen in N,S-CDs was much higher than that in N-CDs, which would result in tend to capture protons from the analytes in the process of desorption. The absorption of N,S-CDs in the near UV region (~355 nm) was stronger than that of N-CDs. CDs and 9-AA as binary matrix for LDI-TOF MS was studied by same group [85]. For the analysis of cytidine, the LOD of the method could down to 5 fmol.

In addition, various noble metals and their composites were utilized as matrices to analyze the biological molecules, such as Au [86,87] and Ag [88] nanoparticles. Typically, Nizioł and co-workers have investigated the gold NPs as matrix for the analysis of the small biological molecules that also can differentiate between normal and cancerous renal tissue [89] and new Ag nanoparticle-enhanced target are used for analysis of a series of biological samples, including urine and blood serum [90]. By using gold NPs (Au NPs) enable the selective capture of thiol-containing compounds, Wan et al. [91] presented a LDI-TOF MS method for fast separation and determination of glutathione (GSH) based on Au NPs as adsorbent and graphene as matrix. The analysis could be performed with less background interference even at the concentration of 0.625 ng/µL. The developed method has been successfully applied to detect GSH in biological samples, such as mouse liver extraction.

Kuo et al. [92] demonstrated a platform based on layer structure of reduced graphene oxide (rGO) and Au NPs as an effective sample plate in LDI-MS. Various small biomolecules were successfully analyzed on the layer structured rGO/Au NPs sample plate with significantly improved signal intensity and reproducibility in comparison to the use of Au NPs or CHCA as the matrices. Silver NPs (Ag NPs) and rGO nanocomposite was fabricated with the LBL electrostatic self-assembly and demonstrated as useful platform for LDI-TOF MS for determination of carboxyl-containing small molecules [93].

Top down synthesized TiO_2_ nanowires were presented as an ideal solid matrix for LDI-TOF MS to analyze small biomolecules by Kim et al. [94]. The TiO_2_ nanowires were synthesized as arrays using a modified hydrothermal process directly on the surface of a Ti plate. The prepared TiO_2_ nanowires in the anatase phase as matrix was successfully applied to analysis of small biomolecules, such as amino acids and peptides. As for analysis of amino acids, the LOD with 10 amol was achieved.

Kailasa and Wu [95] reported a method for one-step preparation of dopamine dithiocarbamate functionalized Au (DDTC-Au) NPs, and utilized as matrix for quantification of small biological molecules (glutathione, desipramine, enrofloxacin, valinomycin and gramicidin D) by LDI-MS. LODs from 0.01 to 1.6 nM with RSDs no more than 6.8% were obtained with the developed method. Except for quantification of small molecules, the as-prepared DDTC-Au NPs were also demonstrated as affinity probes for the enrichment and identification of phosphopeptides from microwave tryptic digests via direct LDI TOF-MS.

Gold-silica (Au@SiO_2_) core-shell NPs (CSNPs) with different core sizes and different shell thicknesses were prepared and used as an energy transfer material for LDI-MS by Zhu et al. [96]. Au@SiO_2_ CSNPs with a smaller gold core (about 18 nm) and ultrathin silica shell (2~4 nm) exhibited the best efficiency including a better signal to noise ratio and signal intensity. SiO_2_@Au core-shell nanomaterials were applied for the analysis of small biomolecules including glucose, cellobiose, phenylalanine, glutamic acid, mannitol and adenosine [97]. By further surface modification with aptamers, Apt-SiO_2_@Au nanoshells allowed simultaneously targeted enrichment and detection of kanamycin with a detection limit at 200 pM.

Due to the unique structure and electronic properties, quantum dots (QDs) were utilized as a matrix for LDI-TOF MS. Bibi and Ju [98] prepared four kinds of QDs including meso-2,3-dimercaptosuccinic acid (DMSA) modified CdTe QDs (DMSA-CdTe QDs), 3,3′-dithiodipropionic acid di(N-hydroxysuccinimide ester) (DSP) QDs (DSP QDs), mercaptopropionic acid (MPA)-stabilized CdS QDs, and thioglycolic acid (TGA)-stabilized CdS QDs. With the help of these QDs, small monosaccharides including arabinose, ribose, xylose, fructose, galactose and mannose, were detected at the amount down to 1 nM with presented sodium and potassium adducts. Six oligosaccharides with the molecular mass ranging from 500 to 700 Da, such as melizitose, isomaltotriose, maltotriose, raffinose, maltoteterose and strachyose tetrahydrate were selected to investigated ionization efficiency, and the results showed that DSP-CdTe QDs as matrix led to the strongest MS signals. The method was successfully used to quantitative analysis of human serum glucose with good reproducibility.

Molybdenum disulfide nanosheets/silver NPs (MoS_2_/Ag) nanohybrid was synthesized and used as a matrix of LDI-TOF MS [99]. The as-prepared nanohybride exhibited high performance for analyzing small biological molecules in negative mode. Compared with pure MoS_2_, the enhancement of LDI efficiency of nanohybrid material could be ascribed to the high surface roughness and large surface area, excellent dispersibility and enhanced thermal conductivity as well as improved UV energy absorption. Moreover, deprotonation sites in both Ag NPs and the edge of the MoS_2_ layers made the nanohybrid materials possessed high ionization efficiency in negative mode.

Lin et al. [100] investigated CuFe_2_O_4_ magnetic nanocrystal clusters (MNCs) as a matrix of LDI-TOF MS for analysis of a series of small biological molecules including peptides, amino acids, nucleobases and fatty acids in negative mode. Bare Fe_3_O_4_ MNCs and MFe_2_O_4_ MNCs (M = Co, Ni, Cu, Zn) were prepared and systematically studied to evaluate they general applicability. Taking analysis of fatty acids for example, MFe_2_O_4_ MNCs (M = Co, Ni, Cu, Zn) could provide 2-to >20-fold signal intensity than that of bare Fe_3_O_4_ MNCs, which indicate the general applicability of using MFe_2_O_4_ MNCs as LDI matrices. The result demonstrated that CuFe_2_O_4_ MNCs was most suitable as matrix and exhibited many advantages including interference-free background, high salt tolerance, and good reproducibility.

Silicon nanopost arrays (NAPA) was demonstrated as effective nanophotonic platforms for LDI of small molecules with rapid, sensitive analysis and minimal sample preparation [101]. Recently, Korte et al. [102] represented a method of LDI-MS from silicon NAPA for large-scale metabolite analysis of metabolite standards (e.g., amino acids, nucleotides, carbohydrates, xenobiotics, lipids, and other classes) and human serum. In untargeted analysis of metabolite standard mixtures, they achieved 374 compounds and useful MS/MS spectra for 287 compounds without individual optimization of ionization or fragmentation conditions. The developed NAPA-based LDI-MS method was found to provide 63% of investigated pathway metabolites in the context of 31 metabolic pathways and 100% of the investigated compounds for the target analysis of the 20 common amino acids. A total of 108 small metabolites and lipids were assigned by direct analysis of aqueous and organic extracts from human serum samples. The method was also successfully applied for the quantitative analysis of glucose and amino acids within the physiological concentration ranges. Besides NAPA, lipid molecules detected by the Au NPs, which exhibits obvious advantages compared with that of conventional MALDI [103].

## 4. Nanomaterial-Assisted LDI for the Analysis of Environmental Pollutants

PAHs as one type of environmental pollutants, ESI-MS that mostly used the ionization method has the difficulty in ionizing them because of their low polarity and stabile chemical properties. Zhang et al. [104] showed that PAHs could be well ionized by LDI with graphene as a matrix. Magnetic graphene nanocomposites were demonstrated as a useful sorbent and matrix for the analysis of nitro-PAHs in PM_2.5_ sample [105]. The LODs with sub pg/μL level were achieved for the detection of three nitro-PAHs (1-NP, 2-nitrofluorene (2-NFL) and 9-nitroanthracene (9-NA)).

Quantitative analysis of nitro-PAHs in PM_2.5_ samples by using LDI-TOF MS with graphene as a matrix was demonstrated by Ma et al. [106]. By using 9-nitroanthracene-*d*_9_ as the internal standard, the LODs of the method for analyzing four typical nitro-PAHs (1-NP, 2-NFL, 6-nitrochrysene (6-NC) and 9-NA) were obtained range from 0.74 to 8.04 ng/µL. The mass concentrations of Σ-nitropyrene, Σ-nitrofluorene, Σ-nitrochrysene and Σ-nitroanthracene on the collected PM_2.5_ samples from haze weather were detected at levels of 0.38 to 3.04 ng/m^3^, 0.21 to 0.43 ng/m^3^, 0.19 to 2.38 ng/m^3^, and 9.55 to 16.52 ng/m^3^, respectively.

Recently, Lu et al. [107] prepared N-doping CDs (N-CDs) and utilized as matrix of LDI-TOF MS for the analysis of the environmental pollutants hydroxyl-PAHs (OH-PAHs) in negative ion mode. Due to the specific π-conjugated polyaromatic structure and the doping of nitrogen atoms, N-CDs as a matrix exhibited low matrix background interference and high signal response. The N-CDs as a new matrix was applied to determine OH-PAHs in real PM_2.5_ samples. With the developed method, the mass concentrations of Σ-hydroxy-pyrene, Σ-dihydroxy-anthraquinone, and Σ-dihydroxy-benzo(α)pyrene on the collected PM_2.5_ samples were determined ranged from 0.125 to 0.136 ng/m^3^, 0.039 to 0.052 ng/m^3^, and 0.053 to 0.072 ng/m^3^, respectively. 

The group of Prof. Jiang did a lot of work on graphene-based materials as matrix LDI-TOF MS for analysis of environmental small molecules [108,109]. To prevent the aggregation of graphene sheets while maintaining the graphene structure as intact as possible, acid-oxidized graphene (AOG) was prepared by oxidizing chemically converted graphene with diluted nitric acid (2 M) [108]. The AOG not only showed good dispersibility in water (up to 1 mg/mL), but also maintained most of the exceptional properties of graphene because the main framework of graphene was not disrupted. Compared with graphene, GO and conventional organic matrix, AOG matrix could provide highest ionization efficiency for nonpolar analytes. Four types of environmental pollutants (pentachlorophenol (PCP), estradiol (E2), 2,2′,4,4′-tetrabromodiphenyl ether (BDE-47), perfluorooctanesulfonic acid (PFOS)) with molecular weight ranged from 266 to 500 Da were analyzed with AOG as matrix. They [109] also reported an improved GO nanoribbons (GONRs) with strong optical absorption and good water dispersibility could be used as a matrix or probe for LDI-TOF MS for simultaneous detection and profiling of multiple small molecules (PCP, E2, BDE-47 and tetrabromobisphenol A (TBBPA) in complex environmental samples. The LODs ranged from 0.8 to 390 pg with RSDs (*n* = 20) lower than 22.3% for above four small molecules were achieved.

rGO film served as the matrix for LDI-TOF MS to analysis of octachlorodibenzo-p-dioxin (OCDD) was demonstrated by Zhou et al. [110]. As little as 500 pg of OCDD could be detected since distinct properties of rGO, such as π-conjugated networks and could facilitate the LDI process. Shi et al. [111] prepared multiwalled CNTs (MWCNTs)@polydopamine (MWCNTs@PDA) core-shell composites and used as matrix of LDI-TOF MS for detection of small molecules. He et al. [112] prepared polystyrene/oxidized CNTs (PS/OCNTs) film by electrospining and used as sorbent of thin film microextraction (TFME) and matrix of LDI-TOF MS for the analysis of BaP from water. The results demonstrated that PS/OCNTs film was a good TFME adsorbent toward the analytes and an excellent matrix for the sensitive determination of BaP and 1-OHP using LDI-TOF-MS. Quantitatively determine BaP in environmental water at concentrations of 50 pg/mL. Applications of magnetic oxidized CNTs or MWCNTs used as adsorbent and matrix of LDI-TOF MS for detection of hazardous organic compounds of BaP and crotonaldehyde were reported by Li et al. [113] and Huang et al. [114], respectively.

To overcome the interference from Au cluster peaks and poor reproducibility, Niu et al. [115] synthesized an Au NP-loaded Zr based MOF by using functionalized amino groups (UiO-66-NH_2_@Au) and used as adsorbent of solid phase extraction (SPE) as well as matrix for LDI-TOF MS. Since the strong affinity to organic pollutants of UiO-66-NH_2_@Au, perfluorinated chemicals including perfluorobutane sulfonate, perfluorohexane sulfonate and PFOS, the endocrine disrupting chemical bisphenol A (BPA), halogenated flame retardants including TBBPA, pentabromophenol and tetrachlorobisphenol A, the pesticide PCP, and hormones including l-thyronine, l-thyroxine and 17-aethynylestradiol at different levels were successfully identified by LDI-TOF MS in negative mode with the assistance of UiO-66-NH_2_@Au.

Ma et al. [25] prepared polydopamine-coated Fe_3_O_4_ NPs (Fe_3_O_4_@PDA NPs) and utilized as matrix for LDI-TOF MS to detection of environmental pollutants. By using Fe_3_O_4_@PDA NPs as matrix, eleven low molecular weight environmental pollutants with molecular weight lower than 500 Da were successfully detected in both positive and negative modes. BaP in real samples (tap and lake water) were detected with Fe_3_O_4_@PDA NPs served as adsorbent and matrix.

**Figure 4:**
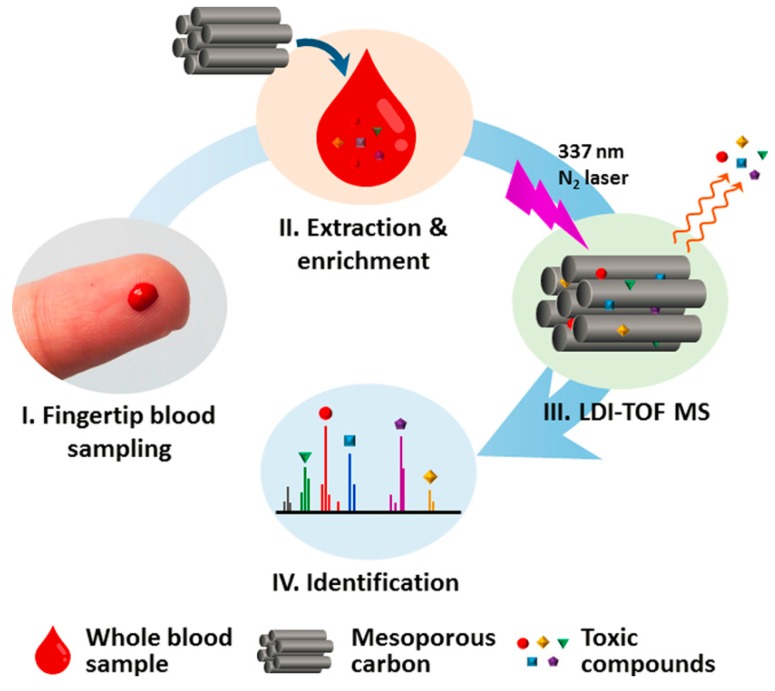
Scheme showing the procedures for the screening of toxic chemicals in a single drop of human whole blood. Reproduced with permission of [116].

Recently, Huang et al. [116] reported a LDI-TOF MS method for screening and identification of toxic compounds in a single drop of human whole blood by using highly ordered mesoporous carbon as both adsorbent and matrix (Figure 4). Among the studied materials including CMK-8, CMK-3, SBA-15, MCM-41, graphene and CHCA, CMK-8 provided the best performance for analysis of target compounds, such as bisphenol S (BPS), TBBPA, BDE-47, PCP, E2 and PFOS. Six perfluorinated compounds in a single drop of whole blood collected from workers in a perfluorochemical plant were screened and identified with high sensitivity (detection limits at ppt level) and good reproducibility by the established method. The results obtained from the developed method were compared with the conventional method (high-performance liquid chromatography-electrospray ionization-tandem MS, HPLC-ESI-MS/MS), due to avoid the complicated sample preparation procedures and can analyze whole blood samples, the developed nanomaterial-assisted LDI-TOF MS method was much faster and more facile than the conventional HPLC-ESI-MS/MS method.

## 5. Nanomaterial-Assisted LDI for the Analysis of Other Small Molecules

TCMs have been widely used in China for the prevention and/or treatment of human diseases for thousands of years. However, compared to modern drugs that contain one or two active compounds with known concentration, TCMs are complex mixtures which usually contain hundreds of chemically different constituents [4]. Therefore, high throughput identification of components (particularly bioactive ingredients) from TCMs becomes the critical factor in the process of “modernization” and “globalization” of TCMs. Liu et al. [117] utilized graphene or GO as matrix of LDI-TOF MS to identify small molecular components TCM herbs. Stable analysis could be achieved without background interference even at the concentration of 100 nM by using graphene or GO as adsorbent and matrix.

Liu et al. [118] reported a graphene-based matrix in LDI-TOF MS at negative ion mode for detection of flavonoids. The results illustrated that signal intensity were higher for flaconoids and the derivatives of coumarin using the GO as the matrix than that of graphene, rGO and 5GO (ultra-large lateral size). It also showed that GO was a more suitable matrix for the analysis of flavonoids.

Antibiotics are commonly used not only in animals for the treatment and prevention of infectious diseases. However, antibiotic residues in the tissues of animals and food (e.g., milk) are inevitable and pose a potential threat to public health. Liu et al. [119] developed a high through LDI-TOF MS method for detection of tetracycline residues in milk by using graphene or GO as an adsorbent and matrix. Tetracyclines were effectively enriched and detected with the LOD as low as 2 nM. Kim et al. [120] synthesized TiO_2_ nanowires with wet-corrosion process and used as matrix for LDI-TOF MS for the analysis of benzylpenicillin in milk. The developed method can be used to analysis of antibiotics in dairy milk samples with LOD of 0.4 ng/mL, which is higher than that of cut-off concentration of the EU directive for antibiotics in milk (4 ng/mL). 

**Figure 5:**
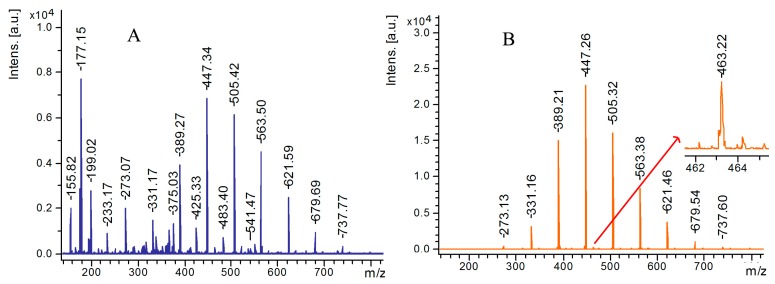
Matrix assisted laser desorption/ionization (MALDI) time-of-flight mass spectrometry (TOF MS) spectra of a low molecular weight polypropylene glycol (PPG) (ca. 425 Da) obtained from analyses by (**A**) MALDI with 2,5-dihydroxybenzoic acid (DHB) as a matrix and (**B**) laser desorption/ionization (LDI) on the layer of graphene nanoparticles (GN). Reproduced with permission of [121].

With the increase in structural and compositional complexity of polymers, characterization of polymer structures was considered as a challenging work. MALDI was considered ideal tool for the analysis of polymers. Due to the interferences from the matrix, it is difficult to use the conventional MALDI method in the analysis of low molecular weight polymers. Lu et al. [121] represented a method for the characterization of polymers by LDI-TOF MS with graphene NPs as assisted-matrix. Taking analysis of polypropylene glycol with average molecular weight of 425 Da for example, mass spectra with strong background interference and interference-free with higher signal intensity were observed with DHB (Figure 5A) and graphene NPs (Figure 5B) as matrices, respectively.

Min et al. [122] prepared gas-phase N-doped graphene (gNG) and applied as a matrix of LDI-TOF MS for small molecule analysis in negative mode. Compared with other graphene-based matrixes, gNG possessed the highly efficiency in desorption/ionization process which can be attributed to the following main factors: highly ordered π-conjugated system and pyridinic-doped nitrogen species. The developed method was successfully applied to detection of anticancer drug nilotinib in the spiked human serum.

Gedda et al. [123] prepared citric acid derived CDs and used as the matrix of LDI-TOF MS for detection of Mefenamic acid (MFA), an anti-inflammatory drug with molecular weight of 241 Da. MAF can be efficiently and sensitively detected by the developed method in positive and negative ion modes with LODs of 0.51 ng and 0.46 ng, respectively. Compared with traditional organic compounds matrix, such as DHB, CDs exhibit extremely high stability to analysis of MFA. The developed method was successfully applied to determination of MFA in human serum even with trace amount.

## 6. Conclusions

We have reviewed the application of a series of nanomaterials, including carbon based materials, metal-based materials and metal organic frameworks, as matrices in LDI-TOF MS for the analysis of small molecules, particularly biological molecules and environmental pollutants, with the publications from 2010. Compared with organic matrices (e.g., CHCA, DHB and SA), nanomaterials as matrices for LDI in the analysis of small molecules provide several advantages. First, serious background interference from conventional organic matrices in low mass region may be eliminated. This feature expands the application of MALDI-TOF MS from large molecules to small molecules. Second, compared to poor shot-to-shot reproducibility in classical MALDI, nanomaterial-assisted LDI provides excellent shot-to-shot reproducibility since the co-crystallization process that usually produces an inhomogeneous mixture and further results in “hot spots” may be avoided. Therefore, nanomaterial-assisted LDI has been demonstrated as a useful method for the quantitative analysis of small molecules. Third, nanomaterials not only can be used as ionization matrix, but also served as adsorbent for pretreatment of complex samples, which enhanced the sensitivity in LDI-MS analysis. Fourth, the competition of analytes and matrix in the ionization process may be eliminated. Thus, nanomaterial-assisted LDI usually offers better signal intensity than classical MALDI in the analysis of small molecules.

Although various nanomaterials have been developed and applied as matrices of LDI for the analysis of different small molecules, there is no systematically study and common theory on how to select proper nanomaterials as matrix for LDI, the key to which is often a matter of trial and error. Nevertheless, it can be concluded that the nanomaterials as a matrix need to meet these basic requirements, including strong adsorption efficiency in ultraviolet light (220–350 nm) range, stable chemical properties, high ability for transfer of absorbed energy to the analytes, and free background signal in the low mass region. As a supplementary technique for the conventional MALDI, nanomaterial-assisted LDI has played considerable roles in the analysis of small biological molecules, environmental pollutants, TCMs molecules, as well as in quantitative analysis.

## Abbreviation

Arg	l-arginine
ATP	adenosine triphosphate
9-AA	9-aminoacridine
BaA	benzo[α]anthracene
BaP	benzo[α]pyrene
BDE-47	2,2′,4,4′-tetrabromodiphenyl ether
BPA	bisphenol A
CD	carbon dots
DHB	2,5-dihydroxybenzoic acid
CHCA	α-cyano-4-hydroxycinnamic acid
CNT	carbon nanotube
ESI	electrospray ionization
E2	estradiol
g-C_3_N_4_	graphitic carbon nitride
Glu	l-glutamine
GO	graphene oxide
His	l-histidine
LBL	layer-by-layer
LDI	laser desorption/inoization
LOD	limits of detection
MALDI	matrix-assisted laser desorption/ionization
MFA	Mefenamic acid
MOF	metal-organic frameworks
Nanomaterial-assisted LDI	nanomaterial-assisted laser desorption/ionization
NAPA	silicon nanopost arrays
NP	nanoparticle
OCDD	octachlorodibenzo-p-dioxin
1-OHP	1-hydroxypyrene
PAHs	polycyclic aromatic hydrocarbons
PCP	pentachlorophenol
PFOS	perfluorooctanesulfonic acid
Phe	l-phenylalanine
ppt,	part per trillion
Rgo	reduced graphene oxide
SA	sinapic acid
SWNHs	single-walled carbon nanohorns
TBBPA	tetrabromobisphenol A
TCMs	traditional Chinese medicines
Trp	l-tryptophan
Tyr	l-tyrosine
9-NA	9-nitroanthracene
2-NFL	2-nitrofluorene
1-NP	1-nitropyrene
